# Estimation of Position Specific Energy as a Feature of Protein Residues from Sequence Alone for Structural Classification

**DOI:** 10.1371/journal.pone.0161452

**Published:** 2016-09-02

**Authors:** Sumaiya Iqbal, Md Tamjidul Hoque

**Affiliations:** Department of Computer Science, University of New Orleans, New Orleans, LA, United States of America; Wilfrid Laurier University, CANADA

## Abstract

A set of features computed from the primary amino acid sequence of proteins, is crucial in the process of inducing a machine learning model that is capable of accurately predicting three-dimensional protein structures. Solutions for existing protein structure prediction problems are in need of features that can capture the complexity of molecular level interactions. With a view to this, we propose a novel approach to estimate *position specific estimated energy* (PSEE) of a residue using contact energy and predicted *relative solvent accessibility* (RSA). Furthermore, we demonstrate PSEE can be reasonably estimated based on sequence information alone. PSEE is useful in identifying the structured as well as unstructured or, intrinsically disordered region of a protein by computing favorable and unfavorable energy respectively, characterized by appropriate threshold. The most intriguing finding, verified empirically, is the indication that the PSEE feature can effectively classify disorder versus ordered residues and can segregate different secondary structure type residues by computing the constituent energies. PSEE values for each amino acid strongly correlate with the hydrophobicity value of the corresponding amino acid. Further, PSEE can be used to detect the existence of critical binding regions that essentially undergo disorder-to-order transitions to perform crucial biological functions. Towards an application of disorder prediction using the PSEE feature, we have rigorously tested and found that a support vector machine model informed by a set of features including PSEE consistently outperforms a model with an identical set of features with PSEE removed. In addition, the new disorder predictor, DisPredict2, shows competitive performance in predicting protein disorder when compared with six existing disordered protein predictors.

## Introduction

Proteins, being the fundamental structural macromolecules of a cell, are involved in most of the cell functions. A fully functional protein is usually the one that is appropriately twisted, coiled and folded into a specific three-dimensional conformation. The three-dimensional structures of proteins specify their associated functions [1, 2]. It is well-known from the literature that the primary protein sequence alone has the essential information needed to determine its corresponding secondary and tertiary structures [3]. However, proteins may misfold under some physicochemical conditions that can alter the usual structural state [4–6]. Moreover, there is an abundance of proteins that are natively unstructured either at the regional level or, sequence wide, known as *intrinsically disordered regions* (IDRs) or, *intrinsically disordered proteins* (IDPs) [5, 6]. IDRs and IDPs become biologically active through disorder-to-structure transitions [4, 7–13]. The connection of IDPs with critical human diseases, such as cancer, cardiovascular diseases, neurodegenerative diseases, genetic diseases, diabetes, amyloidosis and others, has created research areas such as prediction of protein disorder, identification of induced folding region, or binding sites in disordered proteins and drug discovery.

IDPs and protein sequences with IDRs that participate in important biological functions are increasing fast in number [14]. However, the experimental annotation of disordered residues is currently progressing slowly [15, 16]. Thus the computational tools [15–27] for predicting disordered residues using sequence-based features play an alternative and vital role in understanding the functions of disordered proteins and disordered protein regions. These tools that use machine learning algorithms are useful in computational biology as they can produce results quickly. The critical assessment of protein structure prediction, popularly known as CASP competitions [28–30] evaluate the performances of existing disorder predictors biennially since 2002. These predictive tools require a set of features that capture the distinguishing characteristics of the ordered and disordered residues to be predicted. In this article, we propose a novel and effective feature for characterizing different structural descriptors of protein residues from its primary sequence.

Anfinsen’s thermodynamic hypothesis [3] explains that a protein gains the lowest free energy in its natively stable structure. The structural stability of proteins requires a large number of inter-residual interactions. Such interactions among residues result in short range hydrogen-bond formation, van der Waals interactions as well as electrostatic interactions between protein atoms. Inter-residual contact energies between residues in proteins can be estimated from the residue-residue contacts observed in the crystal structures of globular proteins [31, 32]. Attempts have been made to predict the pairwise contact energy values among 20 different amino acids from protein sequence [33]. Energy acts as a measure of proteins’ structural stability. Lower free energy (especially negative energy) is *favorable* for stabilizing the folded state of a protein, whereas an unstable protein gains higher free energy (especially positive energy) that is *unfavorable* for its folded state.

In this work, we introduce a novel approach of predicting the contribution of position specific energy by each residue of a protein sequence to its total energy. We predict this energy per residue from the protein’s primary sequence alone, which we term as *Position Specific Estimated Energy* (PSEE). Note that PSEE does not require a known structure to compute energy, unlike the energy functions [34–36]. The computation of PSEE considers the potential contact partners (amino acids) and the contact energies in the neighborhood of the primary protein sequence as well as the relative exposure of the target residues and its partners. Our empirical results indicate that PSEE can serve as a valuable feature for the prediction of disordered protein, secondary structure types and accessible surface area where 1D sequence information to 3D structural information mapping is essential. As an application, we enhance our disordered protein predictor, DisPredict [16] with the new PSEE feature, named as DisPredict2. DisPredict2 outperforms DisPredict in predicting disordered residues and shows competitive results with six existing disordered protein predictors in the literature.

## Materials and Methods

### Extraction of Position Specific Estimated Energy (PSEE) from Sequence

The free energy of a protein chain is a function of effective inter-residual contacts in its three dimensional conformation. Thomas and Dill described an iterative method [31] to extract interaction potentials, named ENERGI, from a set of protein structures obtained from Protein Data Bank (PDB) [14]. Initially, the 20 × 20 contact energy matrix in [31] is derived from known structures of 37 protein chains. A similar approach is applied in [33] to recalculate the contact energies between all possible pairs of 20 different amino acids using known structures of 785 proteins from PDB. However, the amino acid composition in the primary structure of protein determines its native structure with favorable energy. Therefore, it is believed that the pairwise contact energy can be extracted from the amino acid sequence [33]. The predicted pairwise contact energies in [33] are derived using the primary structures (amino acid sequences) of 674 proteins by least square fitting with the contact energies derived from the tertiary structures of 785 proteins. The actual and predicted energies are found to have a linear relationship, explained in [33].

Here, we present a novel idea of extracting the position specific estimated energy (PSEE) contribution of each residue in a protein from its sequence alone. The preliminary idea behind predicting pairwise energies in [33] conveys that the energy contribution of a residue depends on the amino acid type of that residue as well as the types of its partners in the sequence. We hypothesized that the position specific energy for a protein residue includes the contact effects with different types of amino acids within a neighborhood along the primary sequence. Therefore, we utilize the energy matrix (*P*) derived in [33] and shown in Table 1, to include the effect of having a variable count of different amino acid type residues that can form favorable contacts with the target residue. Further, we hypothesized that the position specific energy contribution of a protein residue is related to the *relative solvent accessibility* (RSA) of the target residue and the residues within its neighborhood region. The RSA of a residue is used to determine its *proportional exposure* (*pExp*) or *proportional burial* (*pBur*) that defines its effective contact surface, therefore can characterize the local environment of that residue in the tertiary structure. In the process of protein folding, the hydrophobic amino acids, having less *pExp*, act as a driving force to develop the core in the tertiary structure, while the hydrophilic residues usually stay on the surface of the protein with relatively higher *pExp*. Thus, *pExp* (or *pBur*) of a residue can provide useful information in capturing the local solvent effects and can help in computing favorable (negative) energy contribution of that residue in its native structure.

**Table 1:** Predicted pairwise contact energy matrix derived in [33].

	A	R	N	D	C	Q	E	G	H	I	L	K	M	F	P	S	T	W	Y	V
A	-1.65	0.98	0.66	1.16	-2.83	1.2	1.8	-0.41	1.9	-3.69	-3.01	0.49	-2.08	-3.73	1.54	-0.08	0.46	0.32	-4.62	-2.31
R	0.98	0.21	1.08	-2.02	-0.41	0.91	-3.13	0.84	0.19	2.05	-0.6	2.34	2.09	-0.4	1.06	0.95	0.98	-5.89	0.36	0.08
N	0.66	1.08	0.61	0.32	-4.18	1.28	0.2	-0.32	1.84	-0.07	0.97	1.12	0.21	0.73	1.15	0.29	0.46	-0.74	0.93	0.93
D	1.16	-2.02	0.32	0.84	-0.82	2.67	1.97	0.88	-1.07	0.68	0.23	-1.93	0.61	-0.92	3.31	0.91	-0.65	-0.71	0.9	0.94
C	-2.83	-0.41	-4.18	-0.82	-39.58	-2.91	-0.53	-2.96	-4.98	0.34	-2.15	-1.38	1.43	-3.07	-2.31	-2.33	-1.84	4.26	-4.46	-0.16
Q	1.2	0.91	1.28	2.67	-2.91	-1.54	0.1	1.11	2.64	-0.18	-0.58	0.43	1.9	0.77	-0.42	1.12	1.65	-2.06	-2.09	0.38
E	1.8	-3.13	0.2	1.97	-0.53	0.1	1.45	1.31	0.61	1.3	1.14	-2.51	2.53	0.94	1.44	0.81	1.54	-1.07	1.29	0.12
G	-0.41	0.84	-0.32	0.88	-2.96	1.11	1.31	-0.2	1.09	-0.65	-0.55	-0.16	-0.52	0.35	2.25	0.71	0.59	1.69	-1.9	-0.38
H	1.9	2.05	1.84	-1.07	-4.98	2.64	0.61	1.09	1.97	-0.71	-0.86	2.89	-0.75	-3.57	0.35	0.82	-0.01	-7.58	-3.2	0.27
I	-3.69	0.19	-0.07	0.68	0.34	-0.18	1.3	-0.65	-0.71	-6.74	-9.01	-0.01	-3.62	-5.88	0.12	-0.15	0.63	-3.78	-5.26	-6.54
L	-3.01	-0.6	0.97	0.23	-2.15	-0.58	1.14	-0.56	-0.86	-9.01	-6.37	0.49	-2.88	-8.59	1.81	-0.41	0.72	-8.31	-4.9	-5.43
K	0.49	2.34	1.12	-1.93	-1.38	0.43	-2.51	-0.16	2.89	-0.01	0.49	1.24	1.61	-0.82	0.51	0.19	-1.11	0.02	-1.19	0.19
M	-2.08	2.09	0.21	0.61	1.43	1.9	2.53	-0.52	-0.75	-3.62	-2.88	1.61	-6.49	-5.34	0.75	1.39	0.63	-6.88	-9.73	-2.59
F	-3.73	-0.4	0.73	-0.92	-3.07	0.77	0.94	0.35	-3.57	-5.88	-8.5	-0.82	-5.34	-11.25	0.32	-2.22	0.11	-7.09	-8.8	-7.05
P	1.54	1.06	1.15	3.31	-2.13	2.97	1.44	2.25	0.35	0.12	1.81	0.51	0.75	0.32	-0.42	1.12	1.65	-2.06	-2.09	0.38
S	-0.08	0.95	0.29	0.91	-2.33	0.85	0.81	0.71	0.82	-0.15	-0.41	0.19	1.39	-2.22	1.12	-0.48	-0.06	-3.03	-0.82	0.13
T	0.46	0.98	0.46	-0.65	-1.84	-0.07	1.54	0.59	-0.01	0.63	0.72	-1.11	0.63	0.11	1.65	-0.06	-0.96	-0.65	-0.37	1.14
W	0.32	-5.89	-0.74	-0.71	4.26	-0.76	-1.07	1.69	-7.58	-3.78	-8.31	0.02	-6.88	-7.09	-2.06	-3.03	-0.65	-1.73	-12.39	-2.13
Y	-4.62	0.36	0.93	0.9	-4.46	0.01	1.29	-1.9	-3.2	-5.26	-4.9	-1.19	-9.73	-8.8	-2.09	-0.37	-0.37	-12.39	-2.68	-3.59
V	-2.31	0.08	0.93	0.94	-0.16	-1.91	0.12	-0.38	0.27	-6.54	-5.43	0.19	-2.59	-7.05	0.38	0.13	1.14	-2.13	-3.59	-4.82

Let, *AA*_*i*_ be the *i*^*t*^*h* amino acid residue of the protein sequence, where *i* ∈ 1,…,*L* and *L* be the length of that protein sequence. *N*_*i*_ is the neighborhood region around *AA*_*i*_ that consists of the contact partner residues of *AA*_*i*_. *N*_*i*_ includes the *contact radius* (CR) number of residues on the either side of a target residue (*AA*_*i*_). Thus the size of *N*_*i*_ is equal to 2*CR*. The predicted pairwise contact energy between *AA*_*i*_ and *AA*_*j*_ is denoted by *P*(*AA*_*i*_, *AA*_*j*_), where *AA*_*j*_ belongs to *N*_*i*_. We weight this contact potential by the proportional burial of the contact partners to capture the essential contact effect in the estimation of position specific energy of the target residue *AA*_*i*_. Therefore, *PSEE*(*AA*_*i*_) is formulated as Eq 1.

$$\begin{array}{c} PSEE\left(AA_{i}\right)=pBur\left(AA_{i}\right)\left[\frac{\sum_{AA_{j}\in N_{i}}P\left(AA_{i},AA_{j}\right)\times pBur\left(AA_{j}\right)}{2CR}\right] \end{array}$$ (1)

#### Computation of proportional exposure (or, burial)

RSA of a protein residue is calculated by normalizing the *accessible surface area* (ASA) of that residue by the surface area of the same type of residue in a reference state. We used the ASA normalizing values derived in [37] using *Gly-X-Gly* tripeptide as the reference state for a given residue X. Therefore, the proportional exposure (*pExp*) and burial (*pBur*) can be expressed by the Eqs 2 and 3.

$$\begin{array}{c} pExp\left(AA_{i}\right)=\left[\frac{predicted\;ASA(AA_{i})}{ASA(AA_{i})\;in\;the\;extended\;conformation\;Gly-X-Gly}\right] \end{array}$$ (2)

$$\begin{array}{c} pBur\left(AA_{i}\right)=1-pExp\left(AA_{i}\right) \end{array}$$ (3)

The ASA normalization values are listed in Table 2. We utilized a new ASA predictor framework, REGAd^3^p [34], to generate predicted ASA of the residues. REGAd^3^p [34] is a new real-value ASA predictor from protein sequence alone that showed maximum Pearson correlation coefficient (PCC) value of 0.76 on a blind test dataset.

**Table 2:** ASA normalization values for 20 amino acids in Å^2^, proposed in [37].

Amino Acid (AA)	ASA normalization value	Amino Acid (AA)	ASA normalization value
Alanine (A)	129.0	Leucine (L)	201.0
Arginine (R)	274.0	Lysine (K)	236.0
Asparagine (N)	195.0	Methionine (M)	224.0
Asparatate (D)	193.0	Phenylalanine (F)	240.0
Cysteine (C)	167.0	Proline (P)	159.0
Glutamine (Q)	225.0	Serine (S)	155.0
Glutamate (E)	223.0	Threonine (T)	172.0
Glycine (G)	104.0	Tryptophan (W)	285.0
Histidine (H)	224.0	Tyrosine (Y)	263.0
Isoleucine (I)	197.0	Valine (V)	174.0

#### Determining contact radius (CR)

PSEE of a residue serves as a measure of the structural stability of that residue being located in that specific position. The structurally stable proteins, so as the residues of proteins, gains energetically favorable (negative) condition compared to the unstructured counterparts. The quantification of PSSE by Eq 1 involves the determination of the contact radius (*CR*) of the neighborhood around the target residue. It is assumed that the target residue forms effective local contacts with the CR number of residues on its either side. To determine the CR parameter for the computation of PSEE, we applied PSEE as a feature to characterize the structured (ordered) and unstructured (disordered) residues. We performed experiments to search for the best CR parameter value in the range of 4 to 30. We executed this experiment on the DisProt database [38] of disordered proteins that stores manually curated annotations of ordered and disordered residues. The recent release of DisProt version 6.02 contains 694 proteins with 1539 disordered regions. We excluded three chains from this set, Id: DP00688, DP00195, DP00642, as they have unknown amino acids, such as X, B and Z. Furthermore, the Cysteine (C) amino acid, being highly reactive due to its sulfhydryl group, caused abnormal PSEE values for some residues of 11 more protein sequences which we have discarded for the aforementioned reason. A very high Cysteine-Cysteine pairwise interaction energy is also explicit in Table 1. Thus we excluded those 11 chains while tuning the value of CR. This purification resulted a list of 680 protein chains, we label as DisProt680 dataset, from DisProt database [38]. Subsequently, we computed the mean PSEE, formulated by Eq 4, of the DisProt annotated ordered (*o*) and disordered (*d*) residues.

$$\begin{array}{c} \bar{PSEE}\left(o\right)=\frac{\sum PSEE(o)}{n_{o}}\;\text{and}\;\bar{PSEE}\left(d\right)=\frac{\sum PSEE(d)}{n_{d}} \end{array}$$ (4)

**Fig 1:**
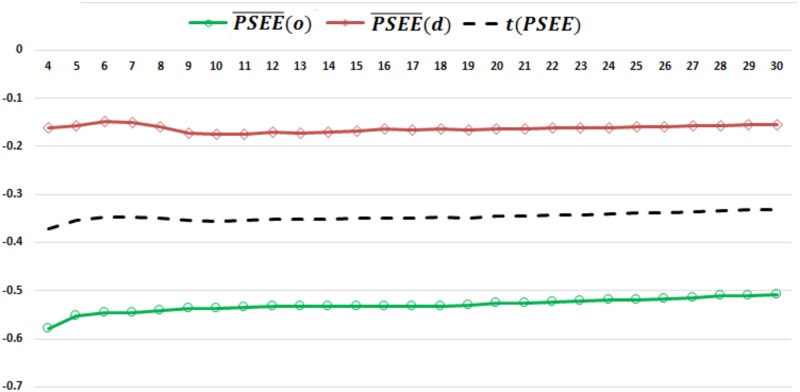
$\bar{PSEE}\left(o\right)$, $\bar{PSEE}\left(d\right)$ and *t*(*PSEE*) for different contact radius (CR) values. Mean PSEE for ordered and disordered residues, indicated by *green line with circle marker* and *red line with diamond marker* respectively, of DisProt680 dataset for CR values of 4 to 30. The separation line or, threshold (*t*(*PSEE*)) is drawn with a black dashed line. The *x*-axis and *y*-axis show the CR and mean PSEE values, respectively.

Here, *n*_*o*_ and *n*_*d*_ are the total number of ordered and disordered residues, respectively. We computed $\bar{PSEE}\left(o\right)$ and $\bar{PSEE}\left(d\right)$ for *CR* values of 4 to 30. For each value of *CR*, we define the threshold, *t*(*PSEE*), for PSEE based identification of ordered and disordered residues as the value that is equally distant from $\bar{PSEE}\left(o\right)$ and $\bar{PSEE}\left(d\right)$. Fig 1 shows the $\bar{PSEE}\left(o\right)$, $\bar{PSEE}\left(d\right)$ and *t*(*PSEE*) for CR from 4 to 30.

**Fig 2:**
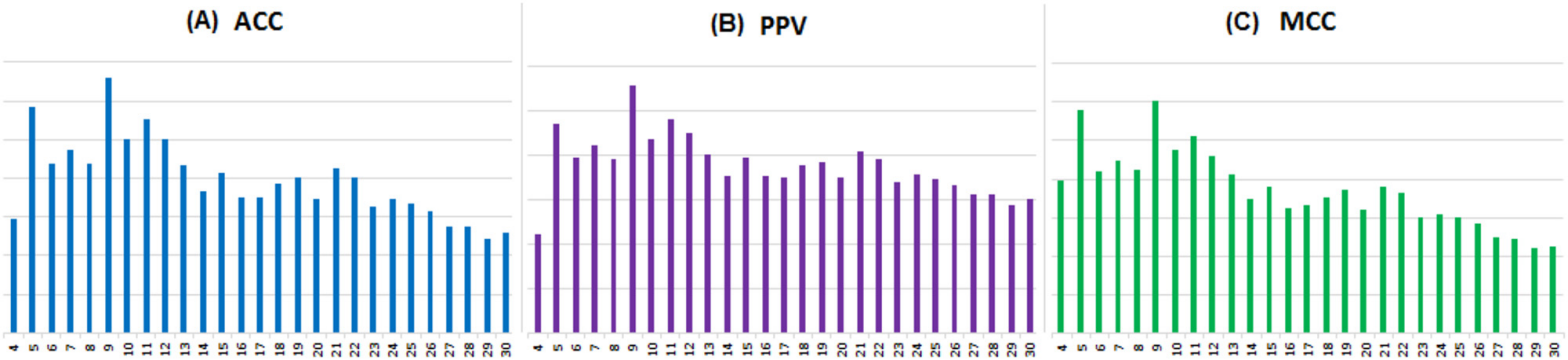
Performance of ordered and disordered residue classification based on per residue PSEE value calculated using different contact radius (CR) values. Classification performance is shown in terms of (A) ACC (*blue bar*), (B) PPV (*purple bar*) and (C) MCC (*green bar*) for CR values equal to 4 to 30. The x-axis and y-axis show the CR values and the performance metric values, respectively.

Fig 1 illustrates that PSEE identifies the energetically induced gap between the structured and unstructured residue and clearly draws the separation line in terms of *t*(*PSEE*) for all values of CR. For CR values equal to 4 to 30, $\bar{PSEE}\left(o\right)$ ranges from -0.51 to -0.58, whereas $\bar{PSEE}\left(d\right)$ ranges from -0.13 to -0.15. Therefore, PSEE could recognize the energetically favorable (negative) condition of structured residues. We utilize *t*(*PSEE*) of the corresponding CR values to classify ordered versus disordered residues to determine the best CR value that most distinguishes PSEE values of ordered and disordered residues. We plot the PSEE based disorder classification performance in terms of *balanced accuracy* (ACC), *precision* (PPV) and *Matthews correlation coefficient* (MCC) in Fig 2. We carried out this preliminary classification based on PSEE only to identify the effective CR value, thus we ignored the actual numerical values of the performance metrics here. Fig 2 shows that PSEE values calculated with a CR value of 9 perform the disordered residue classification most accurately based on the DisProt680 dataset. Thus we obtained the best CR value 9 and we used the same for the rest of our experiments in this work.

### Prediction of Protein Disorder (DisPredict2)

We developed an enhanced disorder predictor, DisPredict2, by incorporating our proposed novel feature, PSEE, into the feature set of our existing predictor, DisPredict [16]. DisPredict2 is available at https://github.com/tamjidul/DisPredict2_PSEE as well as at http://cs.uno.edu/~tamjid/Software/PSEE/PSEE.zip.

#### Datasets

We trained DisPredict2 with the same dataset as was utilized in order to train DisPredict [16] to have an accurate assessment of the effectiveness of the novel feature PSEE. DisPredict2 is trained with 477 protein sequences of the Short-Long (SL) [16, 39] dataset. The SL477 dataset contains protein chains from DisProt [38] database. 50% of the disorder regions in this dataset are short with less than or equal to 20 residues, and the rest are long. The allowable similarity between protein sequence pairs is 25%. SL477 dataset consists of approximately 25%, 34% and 40% of residues annotated as disordered, ordered and unknown. The unknown residues are annotated as ‘X’. We ignored X residues for training and evaluation purposes.

We tested and compared the performance of DisPredict2 with that of DisPredict [16] based on four independent datasets, DD73 [16], CASP8, CASP9 and CASP10. The DD73 dataset was prepared by us and we used it as a holdout dataset in [16]. While the training dataset, SL477, was extracted from the protein chains of the DisProt database version 5.0, DD73 accommodates 48 proteins from DisProt database version 5.1 to 6.02. The rest of the 25 single chain proteins are extracted from PDB [14] with the following criteria: *i*) X-ray structures with resolution ≤ 3.0 Å, *ii*) length *geq* 50 residues, and *iii*) 30% sequence identity cut-off. Later we removed sequences with more than 25% pairwise sequence similarity using BLASTCLUST (ftp://ftp.ncbi.nih.gov/blast/documents/blastclust.html) from the NCBI-BLAST package [40]. Among 73 protein chains, 37 are fully disordered, 23 are fully ordered and 13 have both ordered and disordered regions. For DisPredict2, we utilized the DD73 dataset for both independent evaluation of the predictor and optimization of threshold for disordered residue classification. However, the CASP datasets were kept completely independent, and we did not carry out any optimization on those datasets.

The CASP8 dataset contains 122 protein chains; of which, 103 chains are X-ray derived protein structures and 19 chains are NMR structures. This dataset has approximately 11% disordered residues, and the rest of the residues are structured. We used 111 protein chains of the CASP9 dataset to test and compare DisPredict2 versus DisPredict [16]. For this dataset, only 10% of the total residues were annotated disordered. The CASP9 dataset has a similar proportion of X-ray and NMR derived protein structures. In the CASP10, 94 protein chains were used to assess the disorder predictors. For all CASP datasets, a residue is considered disordered if it lacks spatial coordinates or shows a high conformational variability across different X-ray structures or NMR models.

#### Feature set

In DisPredict2 we appended PSEE to the 56 features per residue used in DisPredict [16] along with PSEE. Therefore, we have 57 features per residue in DisPredict2. The residue level information includes: (*i*) amino acid type, encoded by a single value, as all the necessary information for the correct folding of a protein can be extracted from its amino acid sequence [3]; (*ii*) seven physicochemical properties [41] of amino acids as different types disordered regions (short or long) in a protein are found to have distinguished physicochemical properties; (*iii*) twenty PSSMs (position specific scoring matrix) indicating the evolutionary information conserved in each residue position of a protein sequence; (*iv*) three predicted secondary structure (helix, beta and coil) probabilities from SPINE-X [42], one predicted relative surface area [43] and two predicted backbone torsion angle (phi, psi) fluctuations [35] since disordered residues are characterized by a lack of stable secondary structure, high exposed area and higher fluctuations of torsion angle; (*v*) one monogram and twenty bigrams computed from PSSM [44] representing the conserved evolutionary information in the three-dimensional structural level; (*vi*) one indicator for terminal residues, five residues from N terminal and C terminal are indicated by -1.0 to -0.2 and +0.2 to +1.0 respectively with a step size of 0.2; and (*vii*) one position specific estimated energy (PSEE) value. Finally, before feeding the features into the classifier, 10 neighboring residues’ information, on the either side of the target residue, was aggregated using a sliding window of 21, resulting in 21 × 57 = 1197 features per residue.

#### Predictor framework and performance evaluation

We developed DisPredict2 using the support vector machine (SVM) algorithm, following our initially designed DisPredict predictor [16]. In order to evaluate the contribution of the proposed PSEE feature in Disprodict2, we used the identical parameter optimization and training procedure for the SVM algorithm as in DisPredict [16]. Moreover, the same datasets were used with the PSEE feature appended with the previously used feature set. SVM with radial basis function (RBF) kernel simultaneously minimizes the empirical classification error (training error) and generalized error (test error) by maximizing the geometric margin of the separating hyperplane. The DisPredict2 predictor framework has three levels. The first level is the parameter tuning that determines the optimal values of two parameters for SVM classifier, namely C and *γ*, where C is the cost of misclassification that penalizes the feature space points on the wrong side of the decision boundary and *γ* is the parameter of the RBF kernel. The parameter selection was done by a grid search using 5% of the training dataset, which was guided by 5-fold cross validation with the accuracy (fraction of correctly predicted residues) optimization. The best parameter values found by the grid search is, C = 0.5 and *γ* = 0.0078125. The second level of DisPredict2 development involves the prediction model that generates both binary annotations and real valued probabilities of order versus disorder residues. The probability range, 0.5 ≤ range ≤ 1.0, is considered as disorder probability and 0.0 ≤ range < 0.5 is considered as order probability. The first and second level development of the predictor was done using LIBSVM [33]. The third level of the predictor is to optimize the threshold for disorder classification and to adjust the predicted annotations of each residue based on the optimized threshold. We employed Youden’s J statistic [45] to find the optimal threshold for disorder prediction by analyzing the receiver operating characteristic (ROC) curve using the pROC package [46]. This statistic determines the optimal cut-off that maximizes the distance from the identity (diagonal) line. The optimality criterion is formulated as,

$$\begin{array}{c} max(sensitivities+specificities) \end{array}$$ (5)

To make our predictor robust, we carried out the threshold optimization with an independent test dataset, DD73. The best threshold value found is 0.79. Therefore, we curated the annotation output given by the SVM model using 0.79 ≤ range ≤ 1.0 as disorder probability and 0.0 ≤ range < 0.79 as order probability. Further, we scaled the probability range [0.0, 0.79) into [0.0, 0.5) for the ordered residues and [0.79, 1.0] into [0.5, 1.0] for the disordered residues to make the DisPredict2’s output more natural for binary classification.

The binary outputs given by DisPredict2 is evaluated and compared using the measures listed in Table 3. MCC is considered as the most balanced measure for binary classification [29]. Additionally, we computed *Area Under ROC Curve* (AUC), considered as the measure for the probability assignment. We further plotted the ROC curves and Precision-Recall curves. The AUC values and the both curve plots are generated using the ROCR package [47].

**Table 3:** Name and definition of performance measuring parameters.

Name of metric	Definition
True positive (TP)	Number of correctly predicted disordered residues
True negative (TN)	Number of correctly predicted ordered residues
False positive (FP)	Number of incorrectly predicted disordered residues
False negative (FN)	Number incorrectly predicted ordered residues
Balanced accuracy (ACC)	$\frac{1}{2}\left(\frac{TP}{TP+FN}+\frac{TN}{TN+FP}\right)$
Precision (PPV)	$\frac{TP}{TP+FP}$
Mathews correlation coefficient (MCC)	$\frac{\left(TP\times TN)-(FP\times FN\right)}{\sqrt{\left(TP+FP)(TP+FN)(TN+FP)(TN+FN\right)}}$

## Results

In this section, we highlight the usefulness of PSEE to characterize the structural stability of protein residues. Our results show that PSSE can effectively distinguish ordered and disordered residues, residues of three different secondary structures (helix, beta and coil) as well as residues with different physical properties (hydrophobic and hydrophilic). Therefore, PSEE can effectively extract useful biological information from sequence, making it a useful feature for machine learning based computational tools for disorder prediction, secondary structure prediction, residue exposure prediction, contact prediction, binding region prediction etc. Further, we report the predictive performance of DisPredict2, an updated version of disorder predictor, DisPredict, integrating PSEE into the feature set. The superior performance of DisPredict2 validates the effectiveness of the proposed PSEE feature.

### Discriminatory Capacity of PSEE

#### Ordered and disordered residues

**Fig 3:**
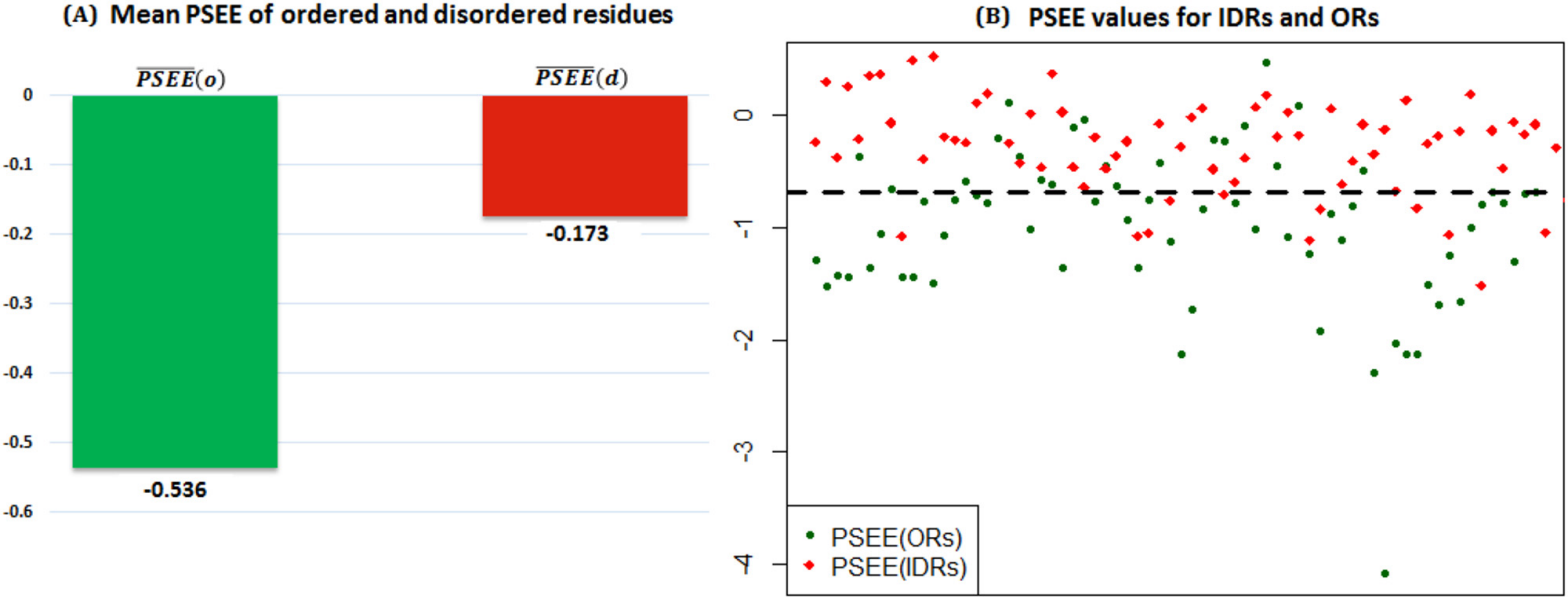
Order versus disorder characterization of PSEE both in residue and region level. (A) Mean PSEE for ordered (*green bar*) and disordered (*red bar*) residues of DisProt680 dataset. The bars are label with the respective mean PSEE values. (B) PSEE values for ordered regions (*green circle*) and disordered regions (*red diamond*). The separation threshold between the average PSEE of all ordered and disordered region is indicated by black dashed line. The *x*-axis and *y*-axis represent the region index and the corresponding PSEE values.

Fig 3(A) shows the mean PSEE of ordered and disordered residues of the DisProt680 dataset with contact radius of 9 on the either side of the target residue. The absolute gap between $\bar{PSEE}\left(o\right)$ and $\bar{PSEE}\left(d\right)$ is 0.363 that makes PSEE a reasonable feature to classify ordered versus disordered residues.

**Fig 4:**
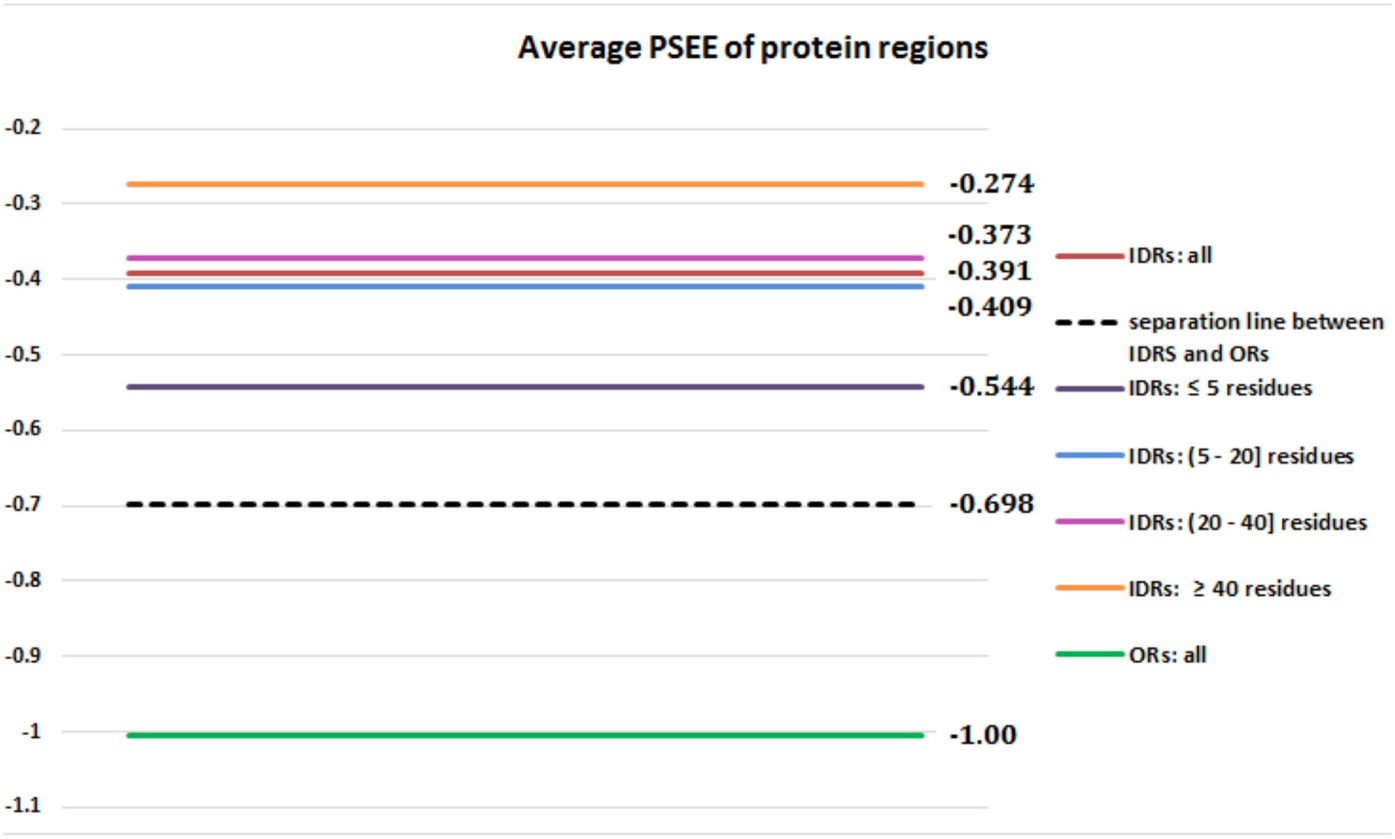
PSEE of different length disordered regions and all ordered regions. Average PSEE of different protein regions of DisProt680 dataset; ORs (*green*), IDRs (red) IDRs with ≥ 40 residues (*orange*), IDRs with (20–40] residues (*pink*), IDRs with (5–20] residues (*blue*), IDRs with *leq* 5 residues (*purple*) and the separation line between all IDRs and ORs is shown by black dashed line. The lines are labeled by the corresponding numerical values of PSEE.

Further, we investigated the PSEE values at the regional level. Fig 3(B) plots the PSEE values for IDRs and ordered regions (ORs) computed as the average PSEE values of the respective residues of the regions. The average PSEE value for all IDRs is -0.391 and that for ORs is -1.00. The black dashed line in Fig 3(B) shows the separation line, computed as the middle value (-0.698) of the two average PSEE values for all IDRs and ORs. Therefore, the region below -0.698 is energetically favorable, whereas above it is the unfavorable region. It shows that PSEE values for some IDRs fall into the favorable region as well. To investigate this further, we segregated the IDRs into four types depending on the length of IDRs; IDRs with ≤ 5 residues, (5–20] residues, (20–40] residues and ≥ 40 residues. Then we computed the average PSEE for all IDRs having similar lengths. Fig 4 shows the average PSEE for all ORs, IDRs, 4 different types of IDRs along with the separation threshold shown in Fig 3(B). The relatively longer IDRs with (20–40] and ≥ 40 residues have PSEE values, -0.373 and -0.274, which are more unfavorable (less negative) than that of considering all IDRs, -0.391. Therefore, PSEE is useful in identifying long disordered regions. It is important to note that the average PSEE for shorter IDRs with ≤ 5 residues, -0.544, is close to the separation line, -0.698, thus these shorter IDRs tend to have favorable energy. These short disordered regions are often called binding sites which are biologically important, as they undergo disorder-to-order transitions by interacting with various partners. Identifying the binding sites in disordered regions is one of the most recent research areas due to the functional importance of binding sites. Our result shows that PSEE values for short disordered regions reflect the usefulness of PSEE in binding site prediction as well.

#### Helix, beta and coil residues

To manifest the performance of PSEE in capturing the structural differences of three different types of secondary structure residues (helix, beta and coil), we computed the mean PSEE for helix (*h*), beta (*e*) and coil (*c*) residues using Eq 6.

$$\begin{array}{c} \bar{PSEE}\left(h\right)=\frac{\sum PSEE(h)}{n_{h}}\text{,}\;\bar{PSEE}\left(e\right)=\frac{\sum PSEE(e)}{n_{e}}\text{,}\;\bar{PSEE}\left(c\right)=\frac{\sum PSEE(c)}{n_{c}} \end{array}$$ (6)

We applied a new secondary structure predictor, called MetaSSPred [48], to generate predicted annotations for helix(h), beta (e) and coil (c) residues. MetaSSPred [48] is a balanced secondary structure predictor that can overcome the under-prediction of less dominating beta residues in the datasets. Helices and beta residues are usually located in the core of the protein, having favorable energy. Beta residues are more structured compared to the helix residues. On the other hand, coil residues stay on the surface areas of proteins and are highly flexible, having unfavorable energy.

**Fig 5:**
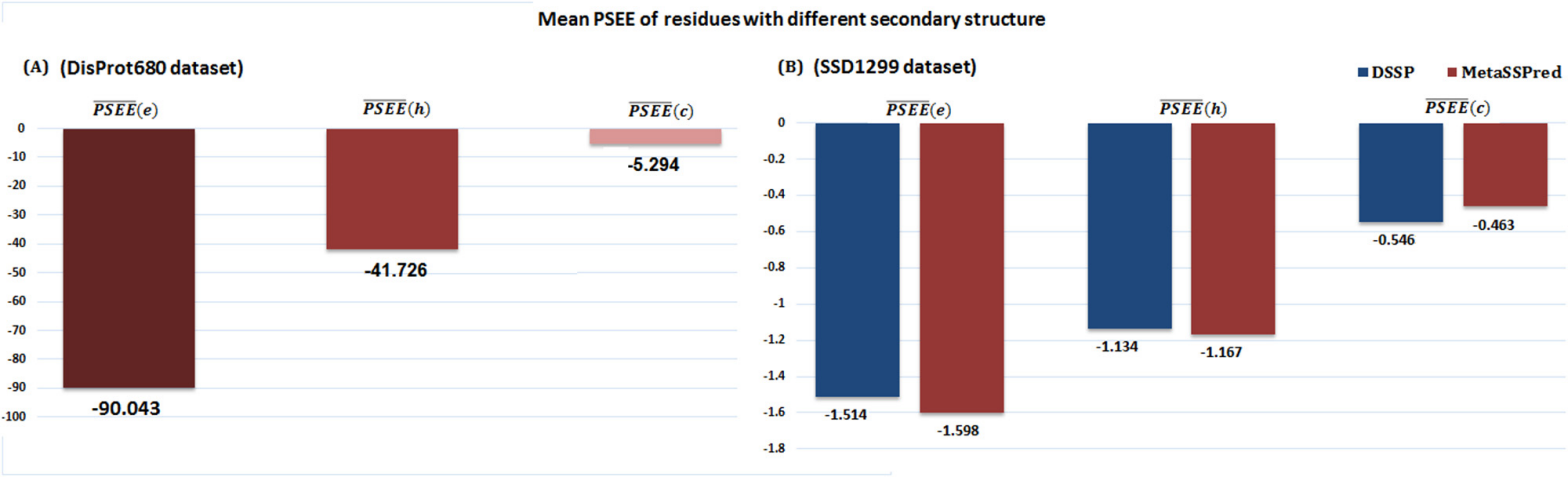
Secondary structure residue type characterization of PSEE. (A) Mean PSEE for beta (dark brown bar), helix (brown bar) and coil (light brown bar) residues of DisProt680 dataset. (B) Mean PSEE for beta, helix, and coil residues of SSD1299 dataset. The blue and brown set of bars represent the annotations from DSSP and MetaSSPred, respectively. The bars are label with the respective mean PSEE values.

Fig 5(A) shows the $\bar{PSEE}\left(h\right)$, $\bar{PSEE}\left(e\right)$ and $\bar{PSEE}\left(c\right)$ for residues of the DisProt680 dataset. Beta residues have the highest negative PSEE and coils possess the lowest negative PSEE, whereas helix residues stay in between the two. This result is reasonable to validate the usefulness of PSEE in identifying different secondary structure residues. To further confirm this, we repeated a similar experiment on another dataset, generated by us in [34], specifically for secondary structure analysis. This dataset is called the secondary structure dataset (SSD) containing 1299 protein sequences with known structures from PDB. We ran DSSP [49] to generate the actual annotations of secondary structures for the residues of the SSD1299 dataset and MetaSSPred [48] for the predicted annotations. The eight class annotations provided by DSSP were converted into three classes using a similar mapping given in [34, 48]. The mean PSEE values for the residues in the SSD1299 dataset are shown in Fig 5(B). PSEE consistently distinguished the three types of residues annotated by DSSP as well as MetaSSPred for the SSD1299 dataset. Therefore, PSEE will serve as a useful feature for secondary structure prediction.

#### Hydrophobic and hydrophilic residues

**Fig 6:**
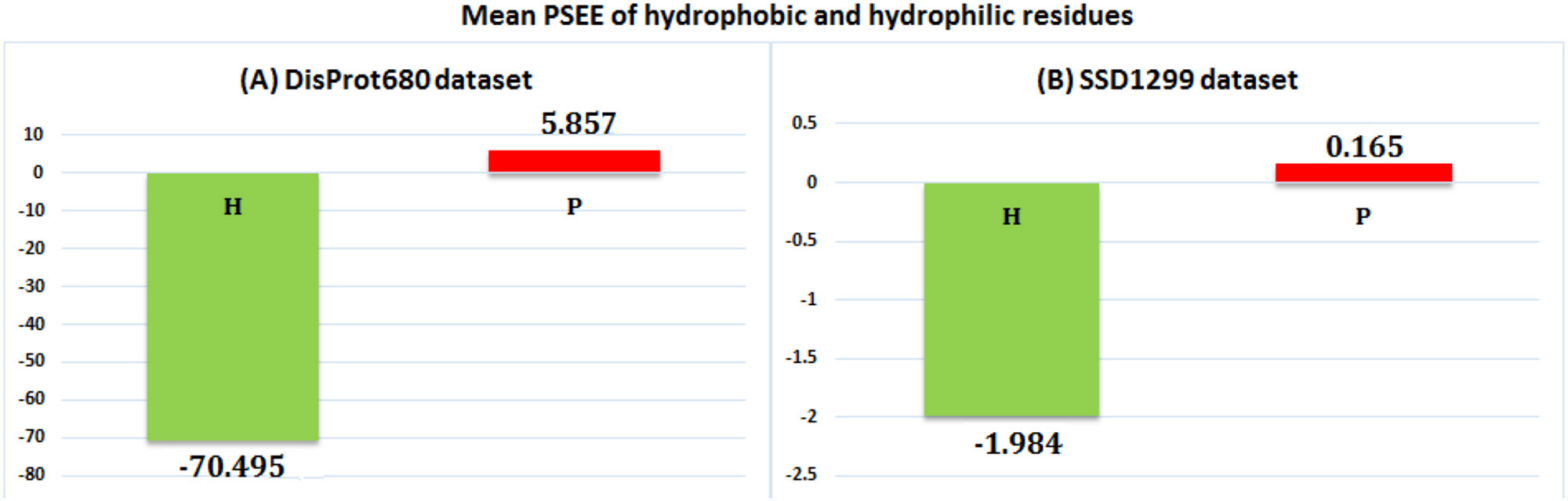
Mean PSEE of hydrophobic and hydrophilic residues. PSEE for hydrophobic (*green bar*) and hydrophilic (*red bar*) residues of (A) DisProt680 dataset and (B) SSD dataset. The bars are label with the respective mean PSEE values.

Hydrophobic (H) amino acids build up the core of a protein and the hydrophilic or Polar (P) ones preferentially cover the surface of the proteins and are in contact with solvent due to their ability to form hydrogen bonds. Therefore, the hydrophobic residues gain energetically favorable conditions compared to hydrophilic residues. Hydrophobic amino acids are A, G, I, L, M, F, P, W, Y and V, whereas the hydrophilic amino acids are R, N, D, C, Q, E, H, K, S and T. We computed mean PSEE for the H and P type residues of both the DisProt680 dataset and the SSD1299 dataset. Fig 6 shows that for both of the datasets, the mean PSEE values for hydrophobic and hydrophilic residues are negative and positive, respectively. Thus PSEE effectively discriminates hydrophobic and hydrophilic residues. As the hydrophobicity of the residues are directly related to the ASA of the residues, PSEE can serve as a useful feature for ASA prediction [34].

**Fig 7:**
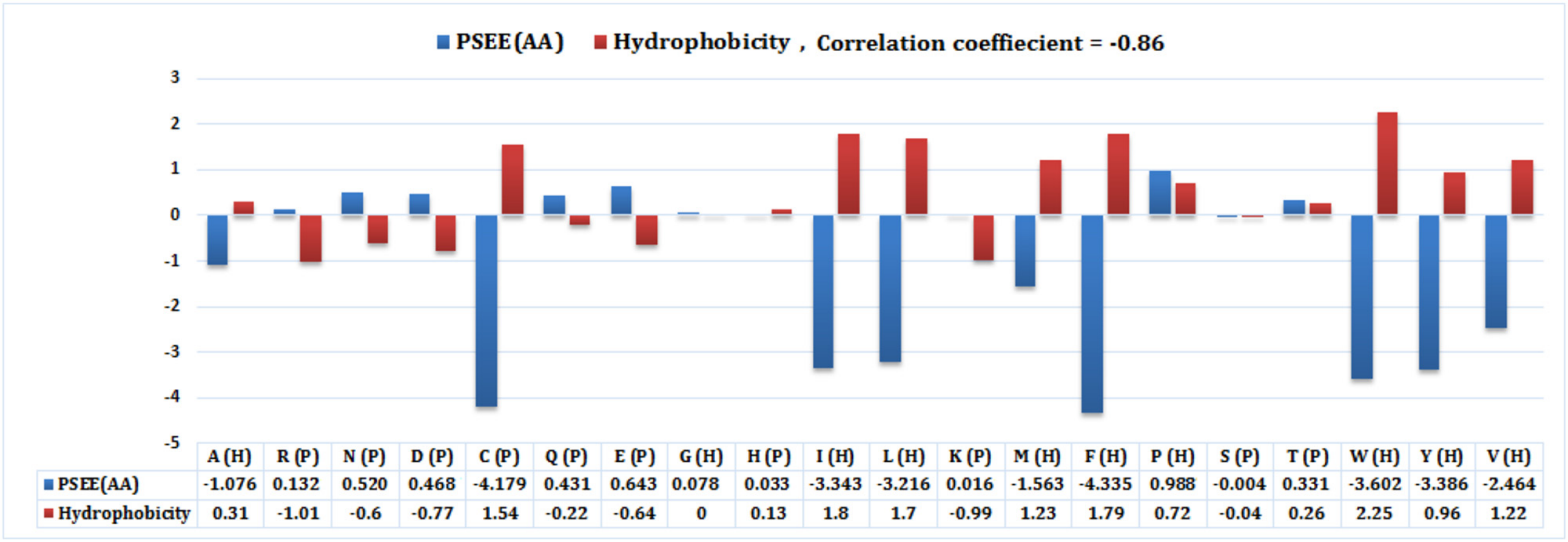
Correlation between mean PSEE and hydrophobicity index of 20 amino acids. Mean PSEE (*blue bar*) and hydrophobicity index (*red bar*) of 20 different types of amino acid residues of SSD1299 dataset. The data values are given in the data table under the plot.

We further collected the hydrophobicity index for 20 different amino acids from [41] and computed mean PSEE for 20 different amino acid residues of the SSD1299 dataset. Essentially the residues with positive hydrophobicity should have a negative mean PSEE. Fig 7 shows the correlation between the hydrophobicity index and the mean PSEE of 20 different amino acid type residues with the correlation coefficient (CC) equal to -0.86. This result emphasizes that (aggregated) PSEE is strongly correlated with the physical property, hydrophobicity, of the amino acid residues, which in turn confirms that the proposed approach is not deviating from the statistics obtained in previous work [41] significantly. A negative CC value is desirable as the high (positive) hydrophobicity of a residue indicates structural stability, thus favorable (negative) energy contribution. Proline is referred as hydrophobic, however it is found more in turns (coils) with unstable structure than helix or beta sheets. Thus it has positive hydrophobicity as well as positive PSEE that correspond to unstable structure.

### Disorder Prediction Performance by DisPredict2

In this section, we measure the benefits of using PSEE as feature in the application of structure (or disorder) classification and prediction in terms of comparing DisPredict2 with 7 other state-of-the-art disorder predictors. These predictors include our initial disorder predictor, DisPredict [16], as well as SPINE-D [19], MFDp [20], MFDp2 [21], Espritz [15], IUPred-Long (IUPred-L) and Short (IUPred-S) [33]. SPINE-D [19] is a two-layered neural network based technique that was initially developed for three state prediction (disordered residues in short and long regions, ordered residue) and later reduced into two state prediction (disordered vs ordered residues). Espritz [15] is a high throughput predictor that uses a recursive neural network. MFDp [20] and MFDp2 [21] are meta predictors that combine different complementary disorder predictors’ output to have further curated prediction. MFDp [20] combines four predicted disorder probabilities from IUPred-L [26, 33], IUPred-S [26, 33], DISOPRED2 [22] and DISOclust [50], while its incremental version, MFDp2 [21], further incorporates sequence based predicted disorder content from DisCon [52]. IUPred-L [26, 33] and IUPred-S [26, 33] predict disordered residues in long and short regions, respectively, using predicted interaction energies. The formulation used in [26, 33] included a sequential local environment by involving interactions with potential partners. Our formulation of PSEE further improvises the pairwise energy based feature by strategically combining the proportional burial information of the potential partners which determines the local structural environment. For a comprehensive comparison, we separately ranked the predictors in terms of balanced accuracy (ACC), Precision (PPV), Mathews correlation coefficient (MCC) and Area Under ROC curve (AUC). We gave the same rank to all predictors having similar scores. We assigned a cumulative score (*S*_*c*_) as a summation of ranks according to different metrics and determined the final rank according to that cumulative score. The results highlight that DisPredict2 is competitive with the different neural network based methods, meta-predictors as well as predictors that uses predicted pairwise energy as a feature. Moreover, the comparative performance analysis of DisPredict2 versus DisPredict is provided to focus the utility of PSEE as a feature for disorder prediction.

**Fig 8:**
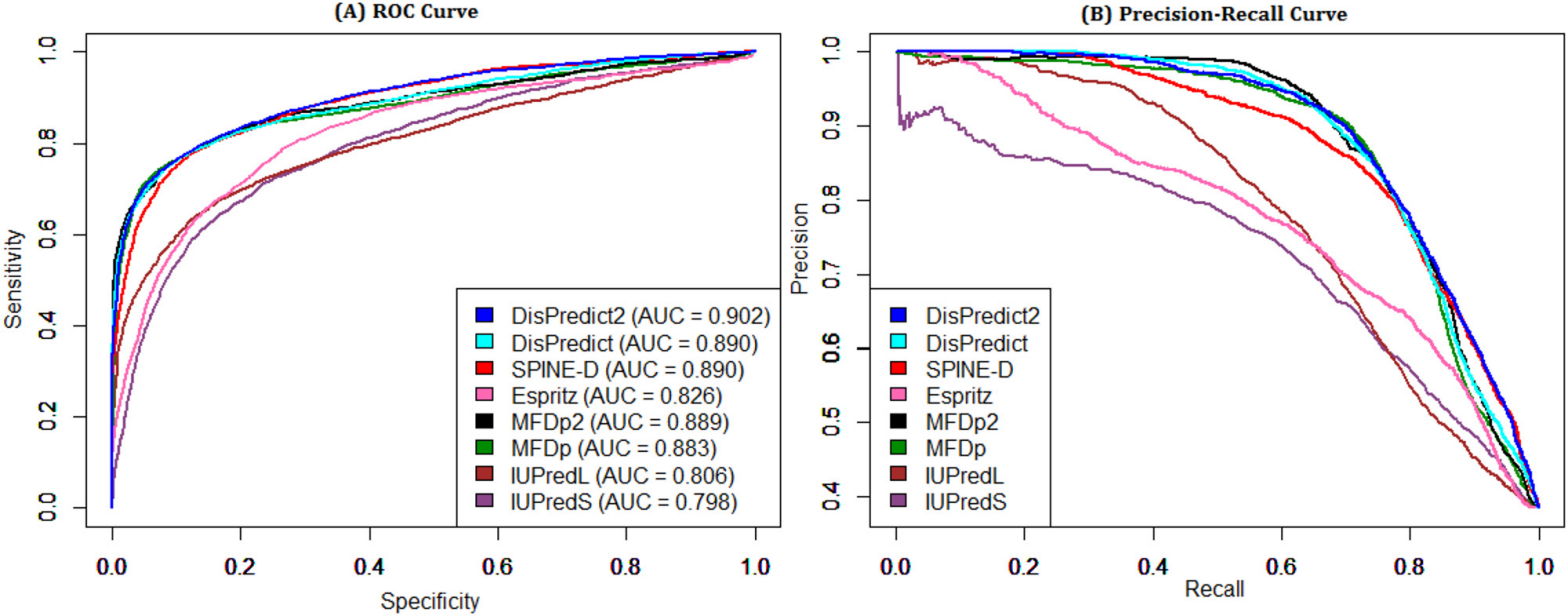
ROC and precision-recall curves given by 8 disorder predictors for DD73 dataset. Comparison of disorder predictors in terms of (A) ROC curve and (B) precision-recall curve on DD73 dataset. The area under ROC curves are given in the plot (A).

Table 4 shows the performance comparison based on the DD73 dataset. This dataset is collected from both DisProt [38] and PDB [14], and is independent from the training dataset, SL477. DisPredict2 was assigned rank 1 in terms of ACC, MCC and AUC as well as achieved highest *S*_*c*_ with a final rank of 1. MFDp2 gave the highest PPV only, however it finally ranked 2 according to the overall performance. Moreover, DisPredict2 provided 0.41%, 6.35%, 3.48% and 1.36% improvement over DisPredict in terms of ACC, PPV, MCC and AUC under the ROC curve, respectively. These improvements focus the benefits of using PSEE as feature. Fig 8 compares the ROC curves and precision-recall curves given by the predictors. DisPredict2, DisPredict and SPINE-D gave comparable ROC curves outperforming the others, while DisPredict2, DisPredict, MFDp and MFDp2 gave better precision for the recall range 0.3 to 0.8 than those of the others.

**Table 4:** Disorder prediction performances of 8 disorder predictors based on DD73 dataset.

Methods	Targets	ACC	PPV	MCC	AUC (ROC)	Ranks				Cumulative Score (*S*_*c*_)	Final Rank
						ACC	PPV	MCC	AUC		
DisPredict2	73	**0.832**	0.857	**0.680**	**0.902**	**1**	2	**1**	**1**	**5**	**1**
DisPredict [16]	73	0.829	0.806	0.663	0.890	2	5	3	2	12	2
SPINE-D [19]	73	0.822	0.766	0.639	0.890	4	8	5	2	19	4
Espritz [15]	73	0.715	0.817	0.494	0.826	7	3	7	6	23	5
MFDp [20]	73	0.828	0.796	0.658	0.883	3	6	4	5	18	3
MFDp2 [21]	73	0.821	**0.873**	0.675	0.889	5	**1**	2	4	12	2
IUPred-L [33]	73	0.742	0.812	0.532	0.806	6	4	6	7	23	5
IUPred-S [33]	73	0.708	0.787	0.471	0.798	8	7	8	8	31	6

Best performances are marked by **bold**.

**Fig 9:**
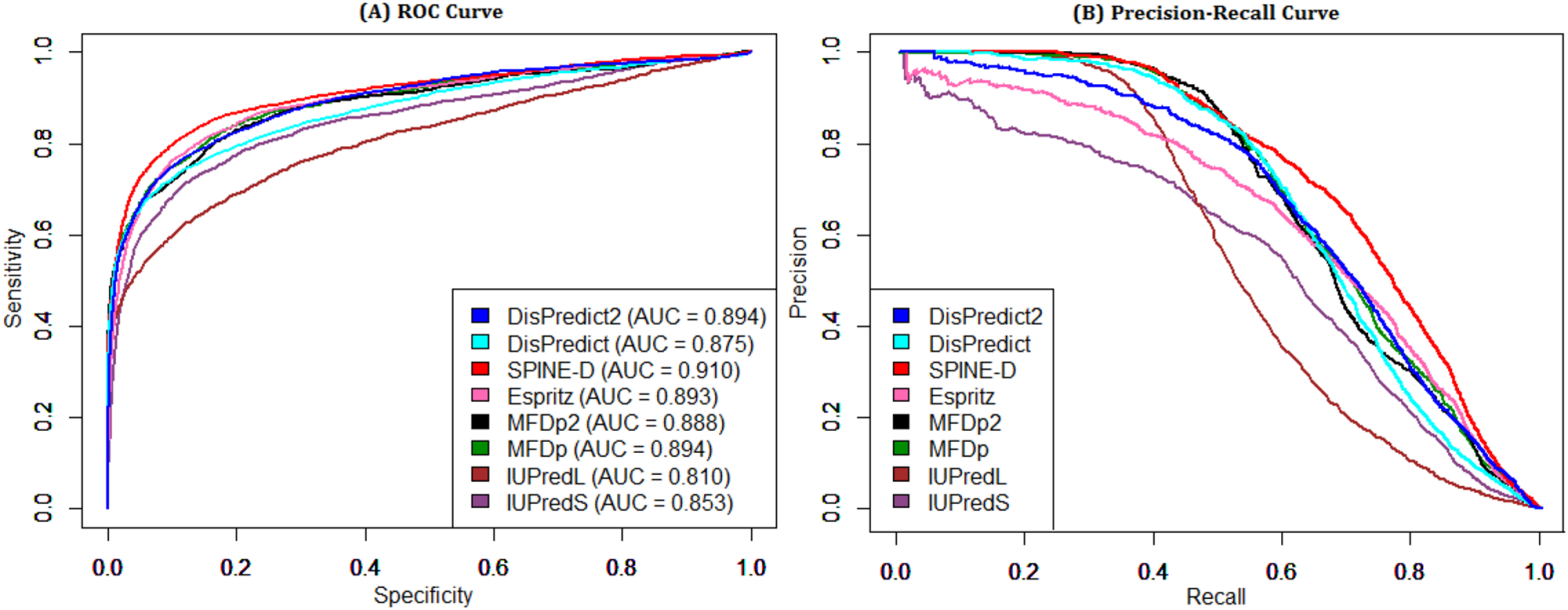
ROC and precision-recall curves given by 8 disorder predictors for CASP8 dataset. Comparison of disorder predictors in terms of (A) ROC curve and (B) precision-recall curve on CASP8 dataset. The area under ROC curves are given in the plot (A).

Table 5 shows the performance of the predictors based on the CASP8 dataset. SPINE-D stood first in terms of ACC and AUC scores, however it gave 33.5% and 7.4% lower PPV and MCC than those MFDp2 whose rank is 1 according to these two scores. DisPredict2 showed comparable performance in terms of all the metrics and attained the best cumulative score, and finally was ranked 1. Thus the overall performance of DisPredict2 is promising. Furthermore, DisPredict2 provided 0.38% lower ACC than that of DisPredict while resulted 18.73%, 8.81% and 2.17% higher PPV, MCC and AUC than those of DisPredict. Fig 9 compares the ROC curves and precision-recall curves. SPINE-D, Espritz, DisPredict2 and MFDp2 gave competitive ROC curves, while the SPINE-D resulted the best precision-recall curve.

**Table 5:** Disorder prediction performances of 8 disorder predictors based on CASP8 dataset.

Methods	Targets	ACC	PPV	MCC	AUC (ROC)	Ranks				Cumulative Score (*S*_*c*_)	Final Rank
						ACC	PPV	MCC	AUC		
DisPredict2	122	0.807	0.628	0.600	0.894	3	5	2	2	**12**	**1**
DisPredict [16]	122	0.810	0.529	0.551	0.875	2	7	6	6	21	6
SPINE-D [19]	122	**0.849**	0.504	0.576	**0.910**	**1**	8	5	**1**	15	4
Espritz [15]	122	0.797	0.636	0.592	0.893	5	3	4	4	16	5
MFDp [20]	122	0.806	0.634	0.601	0.894	4	4	3	2	13	2
MFDp2 [21]	122	0.774	**0.758**	**0.622**	0.888	6	**1**	**1**	5	13	2
IUPred-L [33]	122	0.722	0.700	0.531	0.810	8	2	8	8	26	7
IUPred-S [33]	122	0.766	0.624	0.551	0.853	7	6	6	7	26	7

Best performances are marked by **bold**.

**Fig 10:**
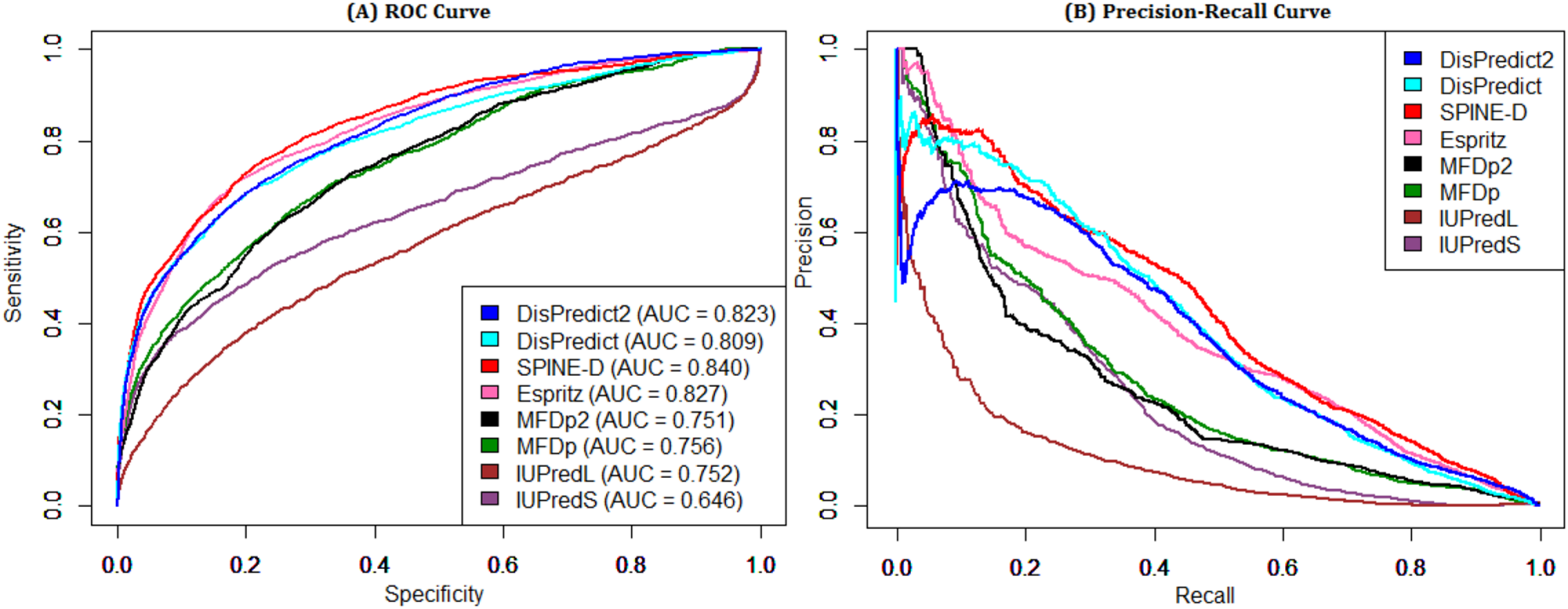
ROC and precision-recall curves given by 8 disorder predictors for CASP9 dataset. Comparison of disorder predictors in terms of (A) ROC curve and (B) precision-recall curve on CASP9 dataset. The area under ROC curves are given in the plot (A).

The comparative performances of the predictors on 111 protein chains of the CASP9 dataset are reported in Table 6. This is a highly imbalanced dataset with approximately 10% of the residues as disordered. MCC is regarded as the best measure in evaluating prediction performance on such an imbalanced dataset as it does not favor the over-prediction of the dominating class [29]. DisPredict2 achieved the best MCC and precision (PPV) score on the CASP9 dataset, while ranked 3^rd^ according to ACC and AUC. Conversely, SPINE-D gave the best ACC and AUC. However, it provided 26.5% lower precision than that of DisPredict2. DisPredict2 obtained the 1^st^ position in the final ranking with cumulative score differences of 2 and 4 from Espritz and SPINE-D respectively in 2^nd^ and 3^rd^ position. Moreover, DisPredict2 with PSEE performed 20%, 5.76% and 1.69% better than DisPredict in terms of PPV, MCC and AUC, respectively; with a slightly lower (2.66%) accuracy. In Fig 10(A) the ROC curves given by DisPredict2, DisPredict, SPINE-D and Espritz show that they were competitive at different points as a result of different thresholds, whereas SPINE-D resulted in the most consistent precision-recall curve. We observed a sharp drop of precision (PPV) in Fig 10(B) at a very low recall value for SPINE-D, DisPredict and DisPredict2. A precision-recall curve essentially plots the PPV and recall scores of a predictor at different threshold values. Therefore, these drops can be the result of having decreasing PPV values (truly positive results out of total positive test outcomes) at some threshold values. However, the PPV values show an increasing trend afterwards.

**Table 6:** Disorder prediction performances of 8 disorder predictors based on CASP9 dataset.

Methods	Targets	ACC	PPV	MCC	AUC (ROC)	Ranks				Cumulative Score (*S*_*c*_)	Final Rank
						ACC	PPV	MCC	AUC		
DisPredict2	111	0.699	**0.471**	**0.407**	0.823	3	**1**	**1**	3	**8**	**1**
DisPredict [16]	111	0.718	0.389	0.385	0.809	2	4	3	4	13	4
SPINE-D [19]	111	**0.745**	0.346	0.385	**0.840**	**1**	7	3	**1**	12	3
Espritz [15]	111	0.683	0.466	0.386	0.827	4	2	2	2	10	2
MFDp [20]	111	0.651	0.361	0.299	0.756	5	6	5	5	21	5
MFDp2 [21]	111	0.616	0.399	0.276	0.751	7	3	7	6	23	6
IUPred-L [33]	111	0.561	0.259	0.147	0.572	8	8	8	8	32	8
IUPred-S [33]	111	0.633	0.466	0.386	0.827	6	5	6	7	24	7

Best performances are marked by **bold**.

**Fig 11:**
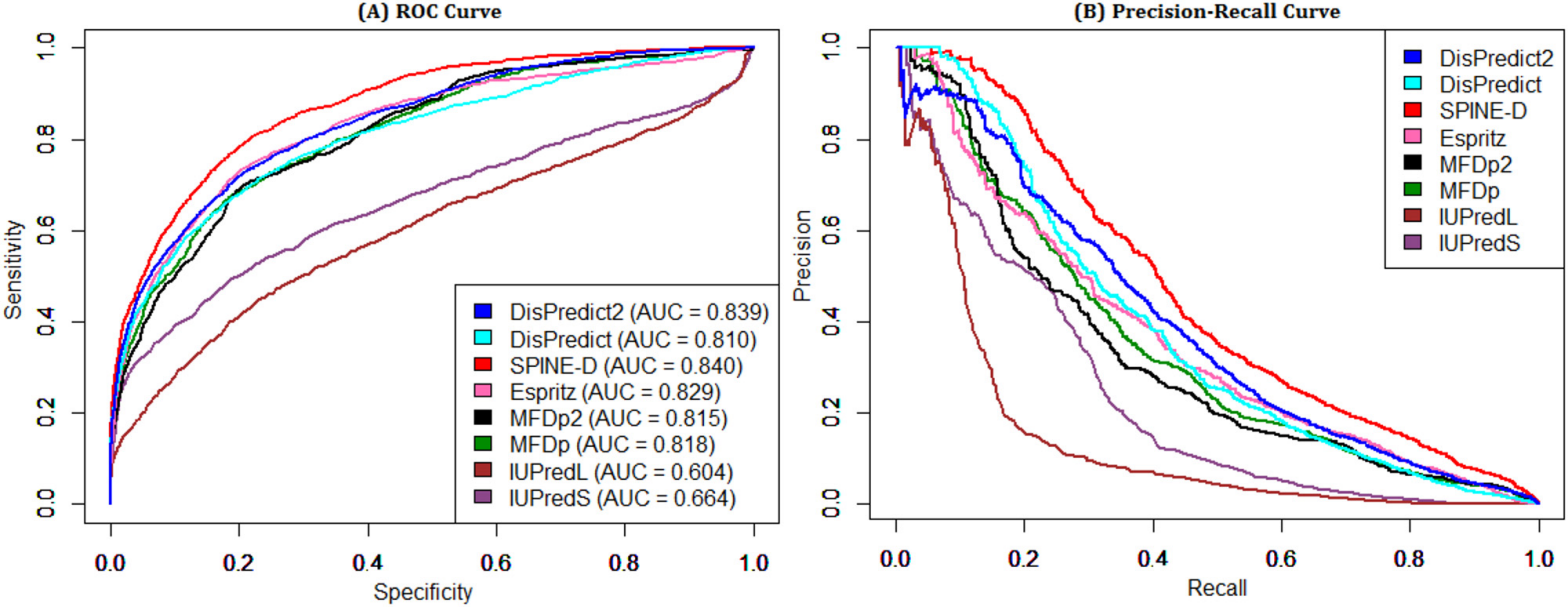
ROC and precision-recall curves given by 8 disorder predictors for CASP10 dataset. Comparison of disorder predictors in terms of (A) ROC curve and (B) precision-recall curve on CASP10 dataset. The area under ROC curves are given in the plot (A).

Table 7 illustrates the performance comparison on the CASP10 dataset. This dataset has only 6.2% of the residues annotated as disordered. DisPredict2 achieved reasonable ranks, however not the best, in terms of all the scores. On the contrary, SPINE-D gave the highest ACC and AUC values with very low precision (ranked 6^th^). Similarly, MFDp2 showed the best precision with low ACC (ranked 6^th^) and Espritz gave best MCC with low ACC (ranked 5^th^). The cumulative ranks of Dispredict2, SPINE-D and Espritz were same, therefore all three of them were finally ranked 1^st^. Moreover, the performance of DisPredict2 is 39.06%, 15.73% and 3.58% higher in terms of PPV, MCC and AUC, respectively. Therefore, DisPredict2 turns out to be a better disorder predictor than DisPredict [16] using PSEE as the only additional features. Fig 11 shows that SPINE-D consistently resulted in better ROC and precision-recall curves with the highest AUC and ACC values in Table 7, whereas the curves of DisPredict, DisPredict2 and Espritz were comparable.

**Table 7:** Disorder prediction performances of 8 disorder predictors based on CASP10 dataset.

Methods	Targets	ACC	PPV	MCC	AUC (ROC)	Ranks				Cumulative Score (*S*_*c*_)	Final Rank
						ACC	PPV	MCC	AUC		
DisPredict2	94	0.719	0.347	0.370	0.839	3	4	2	2	**11**	**1**
DisPredict [16]	94	0.734	0.249	0.320	0.810	2	7	6	6	21	6
SPINE-D [19]	94	**0.774**	0.269	0.366	**0.840**	**1**	6	3	**1**	**11**	**1**
Espritz [15]	94	0.674	0.441	**0.374**	0.829	5	2	**1**	3	**11**	**1**
MFDp [20]	94	0.677	0.359	0.336	0.818	4	3	4	4	15	4
MFDp2 [21]	94	0.636	**0.453**	0.332	0.815	6	**1**	5	5	17	5
IUPred-L [33]	94	0.569	0.238	0.160	0.604	8	8	8	8	32	8
IUPred-S [33]	94	0.635	0.331	0.278	0.664	7	5	7	7	26	7

Best performances are marked by **bold**.

#### Amyloidogenic region (AR) prediction by DisPredict2

**Fig 12:**
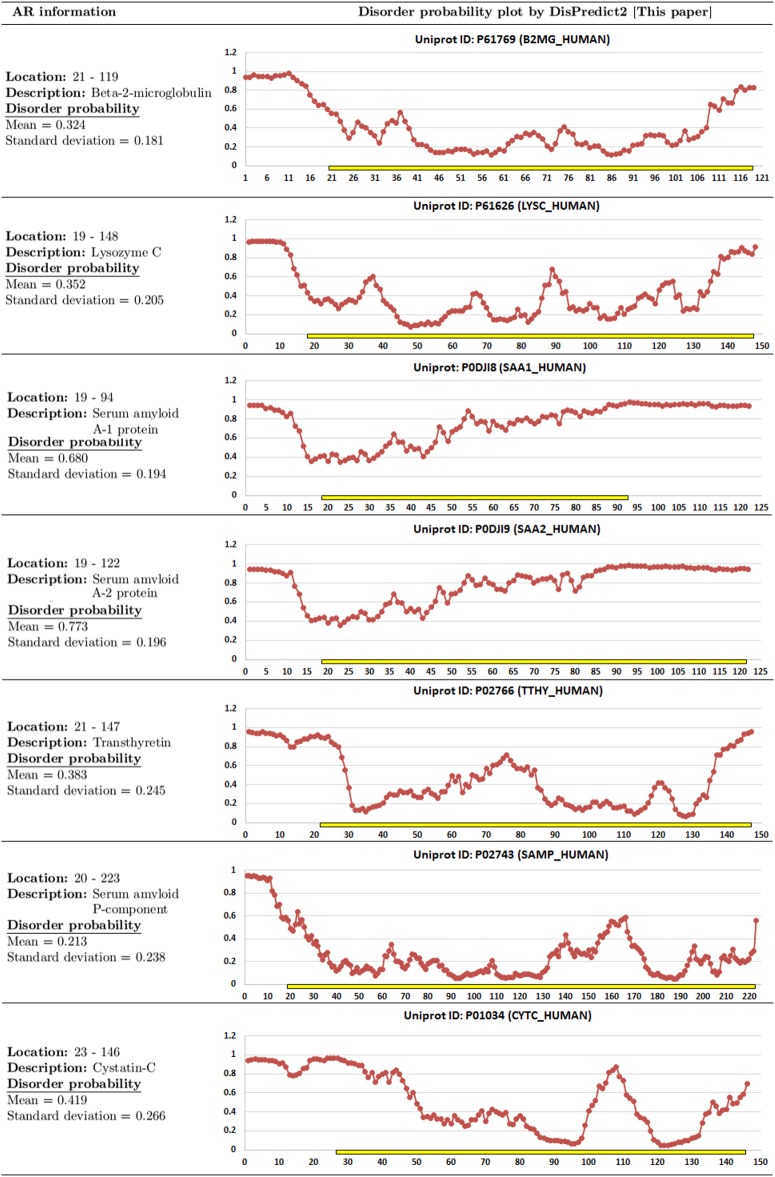
List of proteins with amyloidogenic regions (ARs) and the disorder probability plots given by DisPredict2. The *yellow bar* indicates the ARs and the *red line* shows the disorder probability of each residue indicated by circle marker. The description of protein, location of AR are given on the left side each plot along with the mean and standard deviation of disorder probability for the AR.

To emphasize the biological significance of the outputs provided by DisPredict2, we collected 7 sequences from AMYPdb [51] and computed the disorder probabilities of the residues by DisPredict2. These protein sequences contain amyloidogenic regions (ARs) that are insoluble, however can improperly interact to form amyloids. ARs play an important role in protein aggregation, and they are directly linked with critical human diseases such as neurological disorders. Fig 12 shows the location and description of ARs, mean and standard deviation of disorder probabilities of the residues of ARs, along with probability plot for the proteins. The mean disorder probabilities for seven amyloidogenic regions range from 0.213 to 0.776, with an average of 0.45 (approximately in the middle of the probability range) and standard deviation of 0.203. Therefore, DisPredict2 identified the disorder (without amyloid formation) to order (with amyloid formation) transitions and the associated structural flexibilities of amyloidogenic regions.

## Discussion

In this paper, we describe the extraction of position specific estimated energy, named PSEE, for each residues of a protein based on sequence information alone. The quantification of PSEE includes the interaction effect of the target residue within a neighborhood in terms of pairwise contact energies between different amino acid types. We define the estimated neighborhood size in terms of the number of residues on either side of the target residue with which it can form favorable contacts. Furthermore, it utilizes the predicted relative exposure (or burial) of a residue to approximate the local three-dimensional conformational position and stability of the residue. Our results show that PSEE is very effective in characterizing ordered (structurally stable) and disordered (structurally unstable) residues as well as regions in protein sequences. Moreover, a fine-grained analysis highlights that the average PSEE of the residues of the binding sites in disordered regions are well separable from those of disordered or ordered regions. Therefore, PSEE detects the existence of critical binding regions in disordered proteins that undergo disorder-to-order transitions and perform crucial biological functions [52]. Moreover, PSEE is effective in distinguishing the residues of two different datasets with three different types of secondary structures (helix, beta and coil). The residues with complementary physical properties, such as hydrophobic and hydrophilic, are promisingly identified by PSEE. Moreover, it strongly correlated with the respective hydrophobicity index of 20 different types of amino acids.

**Fig 13:**
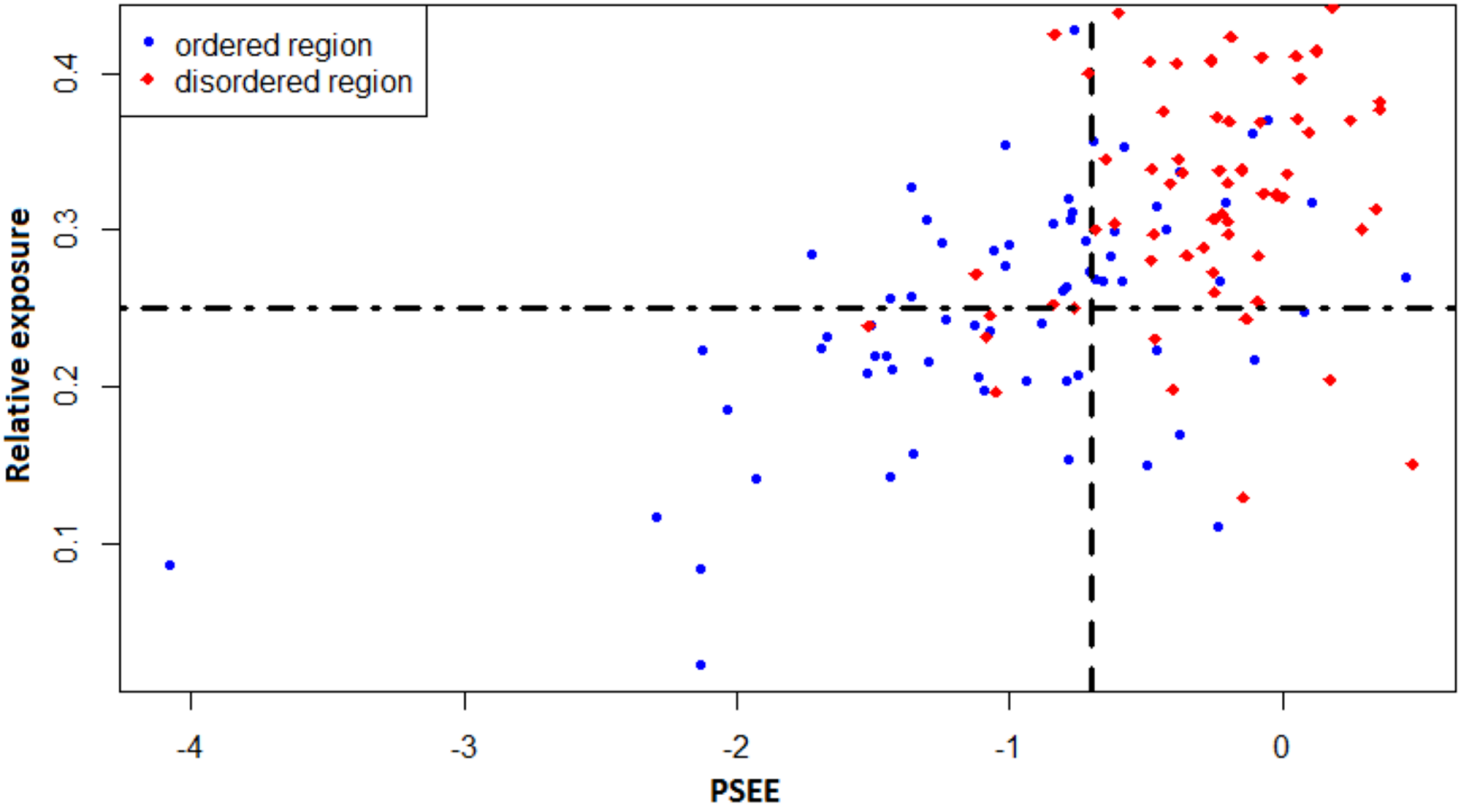
Correlation between PSEE and relative exposure of ordered and disordered regions. PSEE and relative exposure of ordered regions are shown by *blue circles* and those of disordered regions are shown by *red diamonds*. The vertical dashed line separates the average PSEE of ordered and disordered regions and the horizontal dash-dotted line separates the ordered and disordered regions with more and less 25% exposure.

Here, we further discuss the capacity of PSEE to capture multiple structural properties of the residues within the DisProt680 dataset. Fig 13 shows the correlation between *pExp* (or *pBur*) and PSEE of disordered and ordered regions. The vertical dashed line is the separation (-0.698) of PSEE for ORs and IDRs, and the horizontal dash-dotted line indicates separation for exposed or, buried residues. We assumed that the residues with relative exposure less than 25%, computed by Eq 2, are buried. We collected ASA for the residues of the DisProt680 dataset by running REGAd^3^p [34]. Therefore, the left of the vertical line is the energetically favorable regions, and most of the ordered regions (*blue circle*) have PSEE in this region and most of the disordered regions (*red diamond*) have PSEE on the right side. Specifically, the first quadrant (top-right corner) of the plot is the major distribution area of the disordered regions with unfavorable (positive) energy and higher exposure. On the other hand, the third quadrant (bottom-left corner) of the plot is the essential region for ordered regions with favorable (negative) energy and lower exposure. It is explicit in Fig 13 that the PSEE values of most of the disordered regions are in the first quadrant. Therefore, PSEE can capture the exposure-property of the residues and, at the same time, can categorize them as ordered or disordered. However, the other quadrants also contain some disordered regions.

**Fig 14:**
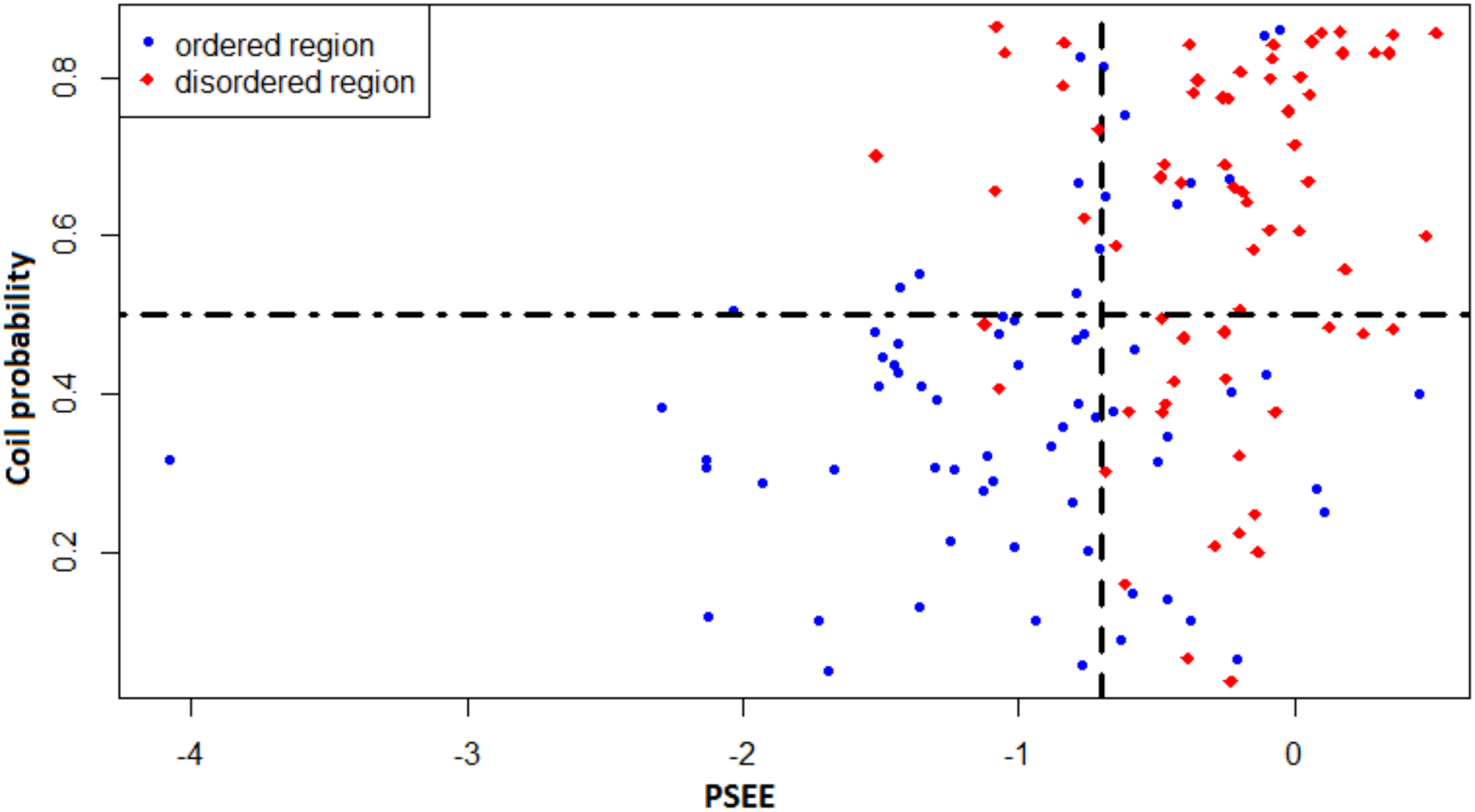
Correlation between PSEE and coil probability of ordered and disordered regions. PSEE and coil probability of ordered regions are marked by *blue circles* and those of disordered regions are marked by *red diamonds*. The vertical black dashed line separates the average PSEE of ordered and disordered region and the horizontal dash-dotted line separates the ordered and disordered regions with more and less 50% coil probability.

Fig 13 shows a similar correlation analysis between the coil-like tendency and PSEE of disordered and ordered regions. We collected coil probability of the residues of DisProt680 dataset by running MetaSSPred [48] and assumed that the residues with higher than 50% coil probability have flexible structure. Therefore, the first quadrant (top-right corner) of the plot is the essential area for disordered regions with unfavorable (positive) energy and high coil probability. On the other hand, the third quadrant (bottom-left corner) of the plot is the essential region for ordered regions with favorable (negative) energy and low coil probability. Fig 14 shows that most of the PSEE values for ordered regions fall in the third quadrant; where those of disordered regions fall in the first quadrant. However, for both Figs 13 and 14, the other quadrants also contain some disordered regions. This can be caused by mis-annotation of disorder [16] from DisProt database or the disorder-to-order transition of binding sites.

This promising correlation among different structural properties and the PSEE of protein residues motivated us to propose PSEE as a feature for the development of predictive tools in the area of bioinformatics and computational biology. To validate our argument, we constructed DisPredict2, a new disorder protein predictor, integrating PSEE in the feature set of an existing disorder protein predictor, DisPredict [16]. DisPredict2 demonstrated improved performance over DisPredict [16] and six other disorder predictors on four different datasets including the CASP8, CASP9 and CASP10 datasets. Moreover, the disorder probability output given by DisPredict2 resembles the flexible structural transformation of amyloidogenic regions of proteins. Therefore, we believe that the new position specific residual feature, PSEE, and the disorder predictor, DisPredict2, both will be effective in understanding several insights of protein structures and their respective functions.

## References

[1] Fischer E. Einfluss der Configuration auf die Wirkung der Enzyme. Berichte der deutschen chemischen Gesellschaft. 1894;27(3):2985–93. 10.1002/cber.18940270364

[2] Petsko GA, Ringe D. Protein structure and function. Protein structure and function; 2004.

[3] Anfinsen CB. The principles that govern the folding of protein chains. Science. 1973;181(4096):223–30. 10.1126/science.181.4096.223 4124164

[4] Wright PE, Dyson HJ. Intrinsically unstructured proteins: re-assessing the protein structure-function paradigm. Journal of Molecular Biology. 1999;193(2):321–31. 10.1006/jmbi.1999.3110 10550212

[5] Uversky VN, Dunker AK. Understanding protein non-folding. Biochimica Et Biophysica Acta (BBA)- Proteins And Proteomics. 2010;1804(6):1231–64. 10.1016/j.bbapap.2010.01.017 20117254 PMC2882790

[6] Uversky VN, Gillespie JR, Fink AL. Why are “natively unfolded” proteins unstructured under physiologic conditions?. Proteins. 2000;41:415–427. 10.1002/1097-0134(20001115)41:3<415::AID-PROT130>3.0.CO;2-7 11025552

[7] Dyson HJ, Wright PE. Coupling of folding and binding for unstructured proteins. Current opinion in structural biology. 2002;12(1):54–60. 10.1016/S0959-440X(02)00289-0 11839490

[8] Tompa P. Intrinsically unstructured proteins. TRENDS in Biochemical Sciences. 2002;10(1):527–33. 10.1016/S0968-0004(02)02169-2 12368089

[9] Dunker AK, Obradovic Z. The protein trinity—linking function and disorder. Nat Biotechnol. 2001;19(6):805–6. 11533628 10.1038/nbt0901-805

[10] Radivojac P, Iakoucheva LM, Oldfield CJ, Obradovic Z, Uversky VN, Dunker AK. Intrinsic Disorder and Functional Proteomics. Biophysical Journal; 2007;92(5):1493–2007. 10.1529/biophysj.106.094045 PMC1796814 17158572

[11] Dunker AK, Brown CJ, Obradovic Z. Identification and functions of usefully disordered proteins. Adv Protein Chem. 2002;62:25–49. 10.1016/S0065-3233(02)62004-2 12418100

[12] Dunker AK, Brown CJ, Lawson JD, Iakoucheva LM, Obradovic Z. Intrinsic disorder and protein function. Biochemistry. 2002;41:6573–82. 10.1021/bi012159 12022860

[13] Uversky VN, Oldfield CJ, Dunker AK. Showing your ID: intrinsic disorder as an ID for recognition, regulation, and cell signaling. J Mol Recogn. 2005;18(5):343–8. 10.1002/jmr.747 16094605

[14] Berman HM, Westbrook J, Feng Z, Gilliland G, Bhat TN, Weissig H, et al The Protein Data Bank. Nucleic Acids Res. 1999;28:135–242. 10.1093/nar/28.1.235 PMC102472 10592235

[15] Walsh I, Martin AJ, Di Domenico T, Tosatto SC. ESpritz: accurate and fast prediction of protein disorder. Bioinformatics. 2012;28(4):503–9. 10.1093/bioinformatics/btr682 22190692

[16] Iqbal S, Hoque MT. DisPredict: A Predictor of Disordered Protein Using Optimized RBF Kernel. PloS One. 2015;10(10):e0141551 10.1371/journal.pone.0141551 26517719 PMC4627842

[18] Iqbal S, Islam MN, Hoque MT. Improved Protein Disorder Predictor by Smoothing Output. International Conference on Computer & Information Technology (ICCIT). 2014;:110–115.

[17] Jones DT, Cozzetto D. DISOPRED3: precise disordered region predictions with annotated proteinbinding activity. Bioinformatics. 2015;31(6):857–63. 10.1093/bioinformatics/btu744 25391399 PMC4380029

[19] Zhang T, Faraggi E, Xue B, Dunker AK, Uversky VN, Zhou Y. SPINE-D: accurate prediction of short and long disordered regions by a single neural-network based method. J Biomol Struct Dyn. 2012;29(4):799–813. 10.1080/073911012010525022 22208280 PMC3297974

[20] Mizianty MJ, Stach W, Chen K, Kedarisetti KD, Disfani FM, Kurgan L. Improved sequence-based prediction of disordered regions with multilayer fusion of multiple information sources. Bioinformatics. 2010;26(18):i489–i96. 10.1093/bioinformatics/btq373 20823312 PMC2935446

[21] Mizianty MJ, Peng Z, Kurgan L. MFDp2: Accurate predictor of disorder in proteins by fusion of disorder probabilities, content and profiles. Intrinsically Disordered Proteins. 2013;1:e24428 10.4161/idp.24428 28516009 PMC5424793

[22] Ward JJ, Sodhi JS, McGuffin LJ, Buxton BF, Jones DT. Prediction and functional analysis of native disorder in proteins from the three kingdoms of life. Journal of molecular biology. 2004;337(3):635–45. 10.1016/j.jmb.2004.02.002 15019783

[23] Ishida T, Kinoshita K. PrDOS: prediction of disordered protein regions from amino acid sequence. Nucleic acids research. 2007;35(suppl 2):W460–W4. 10.1093/nar/gkm363 17567614 PMC1933209

[24] Ishida T, Kinoshita K. Prediction of disordered regions in proteins based on the meta approach. Bioinformatics. 2008;24(11):1344–8. 10.1093/bioinformatics/btn195 18426805

[25] Eickholt J, Cheng J. DNdisorder: predicting protein disorder using boosting and deep networks. BMC bioinformatics. 2013;14(1):88 10.1186/1471-2105-14-88 23497251 PMC3599628

[26] Dosztányi Z, Csizmok V, Tompa P, Simon I. IUPred: web server for the prediction of intrinsically unstructured regions of proteins based on estimated energy content. Bioinformatics. 2005;21(16):3433–4. 10.1093/bioinformatics/bti541 15955779

[27] Kozlowski LP, Bujnicki JM. MetaDisorder: a meta-server for the prediction of intrinsic disorder in proteins. BMC bioinformatics. 2012;13(1):111 10.1186/1471-2105-13-111 22624656 PMC3465245

[28] Monastyrskyy B, Kryshtafovych A, Moult J, Tramontano A, Fidelis K. Assessment of protein disorder region predictions in CASP10. Proteins. 2014;82(Suppl 2):127–37. 10.1002/prot.24391 23946100 PMC4406047

[29] Monastyrskyy B, Fidelis K, Moult J, Tramontano A, Kryshtafovych A. Evaluation of disorder predictions in CASP9. Proteins: Structure, Function, and Bioinformatics. 2011;79(S10):107–18. 10.1002/prot.23161 PMC3212657 21928402

[30] Noivirt-Brik O, Prilusky J, Sussman JL. Assessment of disorder predictions in CASP8. Proteins: Structure, Function, and Bioinformatics. 2009;77(S9):210–6. 10.1002/prot.22586 19774619

[31] Thomas PD, Dill KA. An iterative method for extracting energy-like quantities from protein structures. Proceedings of the National Academy of Sciences. 1996;93(21):11628–33. 10.1073/pnas.93.21.11628 PMC38109 8876187

[32] Miyazawa S, Jernigan RL. Estimation of effective interresidue contact energies from protein crystal structures: quasi-chemical approximation. Macromolecules. 1985;18(3):534–52. 10.1021/ma00145a039

[33] Dosztanyi Z, Csizmok V, Tompa P, Simon I. The pairwise energy content estimated from amino acid composition discriminates between folded and intrinsically unstructured proteins. Journal of molecular biology. 2005;347(4):827–39. 10.1016/j.jmb.2005.01.071 15769473

[34] Iqbal S, Mishra A, Hoque MT. Improved prediction of accessible surface area results in efficient energy function application. Journal of Theoretical Biology. 2015;380:380–91. 10.1016/j.jtbi.2015.06.012 26092374

[35] Zhang T, Faraggi E, Zhou Y. Fluctuations of backbone torsion angles obtained from NMR-determined structures and their prediction. Proteins: Structure, Function, and Bioinformatics. 2010;78(16):3353–62. 10.1002/prot.22842 PMC2976825 20818661

[36] Hoque MT, Yang Y, Mishra A, Zhou Y. sDFIRE: Sequence-specific statistical energy function for protein structure prediction by decoy selections. Journal of Computational Chemistry. 2016 5 5;37(12):1119–24 10.1002/jcc.24298 26849026

[37] Tien MZ, Meyer AG, Sydykova DK, Spielman SJ, Wilke CO. Maximum allowed solvent accessibilites of residues in proteins. PLoS ONE. 2013;8(11): e80635 10.1371/journal.pone.0080635 24278298 PMC3836772

[38] Sickmeier M, Hamilton JA, LeGall T, Vacic V, Cortese MS, Tantos A, et al DisProt: the Database of Disordered Proteins. Nucleic Acids Res. 2007;35:786–93. 10.1093/nar/gkl893 PMC1751543 17145717

[39] Sirota FL, Ooi H-S, Gattermayer T, Schneider G, Eisenhaber F, Maurer-Stroh S. Parameterization of disorder predictors for large-scale applications requiring high specificity by using an extended benchmark dataset. BMC genomics. 2010;11(Suppl 1):S15 10.1186/1471-2164-11-S1-S15 20158872 PMC2822529

[40] Altschul SF, Gish W, Miller W, Myers EW, Lipman DJ. Basic local alignment search tool. J Mol Biol. 1990;215:403–10. 10.1016/S0022-2836(05)80360-2 2231712

[41] Meiler J, Muller M, Zeidler A, Schmäschke F. Generation and evaluation of dimension-reduced amino acid parameter representations by artificial neural networks. J Mol Model. 2001;7:360–9. 10.1007/s008940100038

[42] Faraggi E, Zhang T, Yang Y, Kurgan L, Zhou Y. SPINE X: improving protein secondary structure prediction by multistep learning coupled with prediction of solvent accessible surface area and backbone torsion angles. J Comput Chem. 2012;33(3):259–67. 10.1002/jcc.21968 22045506 PMC3240697

[43] Faraggi E, Xue B, Zhou Y. Improving the prediction accuracy of residue solvent accessibility and realvalue backbone torsion angles of proteins by guided-learning through a two-layer neural network. Proteins. 2009;74:847–56. 10.1002/prot.22193 18704931 PMC2635924

[44] Sharma A, Lyons J, Dehzangi A, Paliwal K. A feature extraction technique using bi-gram probabilities of position specific scoring matrix for protein fold recognition. J Theor Biol. 2013;320:41–6. 10.1016/j.jtbi.2012.12.008 23246717

[45] Youden WJ. Index for rating diagnostic tests. Cancer. 1950;3(1):32–5. 10.1002/1097-0142(1950)3:1<32::AID-CNCR2820030106>3.0.CO;2-3 15405679

[46] Robin X, Turck N, Hainard A, Tiberti N, Lisacek F, Sanchez J-C, et al pROC: an open-source package for R and S+ to analyze and compare ROC curves. BMC bioinformatics. 2011;12(1):77 10.1186/1471-2105-12-77 21414208 PMC3068975

[47] Sing T, Sander O, Beerenwinkel N, Lengauer T, Sing T, Sander O. Visualizing the performance of scoring classifiers. Package ROCR Version 10. 2009;4.

[48] Islam MN, Iqbal S, Hoque MT. A balaced secondary structure predictor. Journal of Theoretical Biology. 2016;389:60–71. 10.1016/j.jtbi.2015.10.015 26549467

[49] Kabsch W, Sander C. Dictionary of protein secondary structure: pattern recognition of hydrogenbonded and geometrical features. Biopolymers. 1983;22:2577–637. 10.1002/bip.360221211 6667333

[50] McGuffin LJ. Intrinsic disorder prediction from the analysis of multiple protein fold recognition models. Bioinformatics. 2008;24:1798–804. 10.1093/bioinformatics/btn326 18579567

[51] Pawlicki S, Le Béchec A, Delamarche C. AMYPdb: a database dedicated to amyloid precursor proteins. BMC bioinformatics. 2008;9(1):273 10.1186/1471-2105-9-273 18544157 PMC2442844

[52] Mészáros B, Simon I, Dosztányi Z. Prediction of protein binding regions in disordered proteins. PLoS Comput Biol. 2009;5(5):e1000376 10.1371/journal.pcbi.1000376 19412530 PMC2671142

